# A Home-Based Multimedia Pulmonary Rehabilitation Program Improves Clinical Symptoms and Physical Performance of Patients with Chronic Obstructive Pulmonary Disease

**DOI:** 10.3390/ijerph182111479

**Published:** 2021-10-31

**Authors:** Anoma Santiworakul, Nitita Piya-amornphan, Netchanok Jianramas

**Affiliations:** Department of Physical Therapy, School of Allied Health Science, Walailak University, 222 Thasala, Thaiburi, Nakhon Si Thammarat 80160, Thailand; panoma@wu.ac.th (A.S.); nitita.do@wu.ac.th (N.P.-a.)

**Keywords:** home-based pulmonary rehabilitation, chronic obstructive pulmonary disease, exercise capacity

## Abstract

Home-based pulmonary rehabilitation can decrease symptoms in chronic obstructive pulmonary disease (COPD) patients. The purpose of this study was to compare the effects of a home-based pulmonary rehabilitation by instructive multimedia in the form of videos and flip charts on clinical symptoms and exercise performance in COPD patients. An eight-week home-based pulmonary rehabilitation program was performed with twenty COPD patients older than 60 years of age with moderate to severe stages. They were separated into two groups: a multimedia group (*n* = 10) and a control group, which was only provided with telephone monitoring (*n* = 10). Clinical symptoms were measured by using the clinical COPD questionnaire (CCQ), and exercise performance was measured using a six-minute walk test (6MWT) and an upper-lower limb muscle strengthening test. After 8 weeks, the results showed that both groups showed a statistically significant decrease in the CCQ *(p* < 0.05). The multimedia group showed a statistically significant increase in the lower-limb muscle strengthening *(p* < 0.05), while the control group was not found to show a statistically significant increase in the lower-limb muscle strengthening. Therefore, a pulmonary rehabilitation program using multimedia at home can lessen symptoms and improve exercise performance in COPD patients.

## 1. Introduction

Chronic obstructive pulmonary disease (COPD) is one of the world’s chronic non-communicable diseases [1,2]. Patients with COPD are found throughout the world, in both urban and rural areas [3]. They have chronic inflammation of the airways, resulting in decreased air entry into the lungs, shortness of breath, and a reduced ability to carry out daily activities [4,5]. The multidisciplinary program of care, involving both pharmacological and nonpharmacological methods, improves the patient’s condition by decreasing dyspnea, fatigue, and anxiety regarding health issues, as well as by increasing exercise capacity and quality of life. These bring about long-term care costs for both the government and the patient’s family [6,7].

Pulmonary rehabilitation (PR) for patients with chronic obstructive pulmonary disease, including exercise training, education for smoking cessation and nutrition, self-management, and/or behavioral change programs, aim to improve the physical and psychological conditions of COPD patients [1]. A home-based pulmonary rehabilitation program is important in minimizing issues for rural patients who have difficulty with transportation from isolated locations or epidemic conditions that require social distancing, such as COVID-19 [8,9]. Home-based pulmonary rehabilitation is safe and improves clinical outcomes, in addition to reducing the cost of patient care. Previous studies support the impact of home-based rehabilitation and that it is not lower than center-based pulmonary rehabilitation [10,11]. There are various models of pulmonary telerehabilitation, including home videoconferencing, a combination of videoconferencing and telephone support, and web-based self-monitoring. A 6-week online pulmonary rehabilitation program showed positive effects in terms of a six-minute walk test (6MWT) distance and symptom scores [12]. An interactive web-based PR program showed increased endurance in the shuttle walk test and improved Chronic Respiratory Disease Questionnaire Dyspnea (CRQ-D) score [11]. The programs were safe, well-tolerated, and acceptable when compared with hospital-based pulmonary rehabilitation [11,12]

However, previous studies identified technical and personal concerns when choosing a method of telerehabilitation program. Survey researchers found that some COPD patients have problems with slow internet speed, poor network signal, system complexity, feeling too old to adopt new technology, difficulty navigating or installing the system on their computer, and lack of patient training and knowledge regarding technology, which represents barriers to people using telerehabilitation [8,13]. In remote areas, communication infrastructure, including internet signal distribution, accessibility to technology, and ability to use technology, was not adequate. Offline and telephone-based home pulmonary rehabilitation may be a solution. In this case, informative media may be important, especially in COPD patients with cognitive impairment, who show a higher incident rate than the general population [14,15]. The cognitive impairment may affect the patient’s medical attention and treatment regimens by increased confusion and memory loss, which impact the patient’s daily function [16,17]. Pulmonary rehabilitation programs should be concerned with education, support, and self-management according to the objectives of the program for behavioral changes and the enhancement of a patient’s health. Patients have different learning styles and attention spans; therefore, the use of multiple modalities is preferred [18,19]. Multimedia in the form of videos, and flip charts for education and exercise on home-based pulmonary rehabilitation, for all learning preference stimulating perception, including visual, auditory, reading, and kinesthetics of patients, is feasible and accessible for elderly COPD patients with barriers to online communication.

A previous study studied the effect of a home-based pulmonary rehabilitation program using a booklet. Their program included exercises, plus an explanation about the disease and the importance of physical exercise to the quality of life. They reported that the walked distance, the maximal inspiratory pressure, and the health-related quality of life were increased after the 8-week program [20]. Another study involving home-based rehabilitation using a manual, which included respiratory exercises, upper- and lower-limb exercises, step training, and strategies to control dyspnea, showed its effectiveness in maintaining the benefits acquired during the outpatient rehabilitation program [21]. From previous studies, they used the instructional format of pulmonary rehabilitation programs, including exercise and related education, mainly stimulated via reading and kinesthetic learning preferences. However, to facilitate COPD patients in engaging with a multidisciplinary home-based pulmonary rehabilitation program, an instructive multimedia format for facilitating all learning preferences of patients should be investigated. This study aimed to determine the effect of a multidisciplinary home-based rehabilitation program, including exercise training, education in nutrition, medication management, smoking cessation, and self-management using instructive multimedia in the form of videos and flip charts on clinical symptoms, exercise capacity, and strengthening of the muscles of the upper and lower extremities in patients with chronic obstructive pulmonary disease.

## 2. Materials and Methods

### 2.1. Study Design and Setting

This study employed a randomized control trial (RCT). According to the number of research participants in the pilot study, an independent-sample *t*-test determined an α-error of 0.05 and a β-error of 0.20. Each sample group contained 11 people. The inclusion criteria included chronic obstructive pulmonary disease, patients aged ≤60 years, whose condition was moderate to severe (30% ≤ FEV_1_ < 80% predicted [1]), who were without malnutrition (BMI ≥ 18.5 kg/m^2^), and who had continuously received treatment for at least 1 year. The exclusion criteria were as follows: patients who had cardiovascular or neurological diseases; patients who had fallen down or had a history of broken bones; patients with other systemic diseases, such as lung cancer, infection, anxiety and depression, or diabetes; patients with hypertension who did not receive regular medicine or abstained from treatment for more than 6 months; patients with a condition made unstable by the rehabilitation program; chronic obstructive pulmonary disease patients with an exacerbated condition who had received hospital treatment due to dyspnea more than three times in the past year.

### 2.2. Data Collection

The participants were divided into two groups by simple randomization: (1) an experimental group, which received multimedia pulmonary rehabilitation with videos and flip charts, as well as structured telephone monitoring, and (2) a control group, which received pulmonary rehabilitation via structured telephone monitoring only. During the first visit, the participants had their vital signs checked, i.e., heart rate (HR), respiratory rate (RR), blood pressure (BP), and oxygen saturation (SpO_2_). The participants were requested to complete the Montreal Cognitive Assessment (MoCA), which is an evaluation to identify patients with learning, understanding, and memory impairments. A clinical COPD questionnaire (CCQ) self-evaluation [22] was completed to evaluate muscle strength using a handheld dynamometer measuring the arm muscle strength by maximum isometric contraction at a 90° angle, as well as leg muscle strength by maximum isometric contraction with three repetitions at a 60° angle. The participants were requested to take a break between measurements to prevent fatigue before recording and reporting the best result [23]. Then, workout ability was evaluated using a 6MWT following established guidelines. The participants had their vital signs checked before and after [24]. During the second and third visits, the participants were reassessed in terms of CCQ, muscle strength, and 6MWT to determine the outcome of the program.

### 2.3. Intervention

Participants in both groups received the core components of pulmonary rehabilitation, including self-management education, aerobic exercise training, and resistance training. This program consisted of watching a 38-min guidelines video (self-management video) about smoking cessation from a registered nurse, nutrition of five food groups, along with food to eat and food to avoid, from a nutritionist, and correct medical use from a pharmacist. A physical therapist instructed the patients on breathing exercises, aerobic exercises, resistance exercises, and chest trunk mobilization. Both participant groups watched the self-management video along with a physiotherapist at the hospital and addressed any questions that arose. Participants in the multimedia group watched an exercise video and were shown how to perform the exercises in the video (Table 1) at home at least three times per week for a period of 8 weeks. The exercise video consisted of a warmup (stretching upper and lower limbs), resistance exercise (elastic band), aerobic exercise, and cooldown. The control group received the program from a physiotherapist, which contained content similar to the video. The exercise progression of both groups could be adjusted every week after the telephone monitoring with a physiotherapist by increasing the number of cycles of on-the-spot marching alternated with chest trunk mobilization. At week 4, The subject demonstrated the exercise with a physiotherapist and increased the intensity of the aerobic exercise by increasing the speed of on-the-spot marching and/or increased the number of cycles of on-the-spot marching alternated with chest trunk mobilization (within Borg scale 3–4) [25]. For the resistance exercise, the participants adjusted the range of an elastic band when they did a resistance exercise of more than 10 RM.

In both groups, the patient’s family members facilitated the uptake of program information and were invited to help participants during home pulmonary rehabilitation. Structured telephone monitoring, conducted for 10–20 min, once per week, was used to evaluate patient symptoms, stimulate motivation, review goals of the pulmonary rehabilitation program, record the participant’s exercise program, and address problems with the program or patient needs [26]. A physiotherapist was trained to carry out the structured telephone monitoring, which was recorded for review with the pulmonary rehabilitation team.

### 2.4. Statistical Analysis

Statistical analysis was carried out using SPSS (Statistics Package for the Social Science) version 21. Statistical significance was determined at *p* < 0.05. The normal distribution of data was tested using the Kolmogorov–Smirnov goodness-of-fit test. An independent *t*-test was used to compare the characteristics between groups. Two-way mixed ANOVA was used to compare the differences between groups in a clinical COPD questionnaire (CCQ) score, 6MWT distance, and upper- and lower-limb muscle strength at weeks 4 and 8. Bonferroni correction was used for post-hoc analysis.

### 2.5. Ethical Considerations

This study was approved by the Institutional Review Board of the Ethics Committee of Human Ethics, Walailak University (WUEC-18-128-01). The participants were given a detailed description of the study’s purpose, methods, and potential benefits of participation, as well as a privacy statement. Data collection was commenced after the participants signed informed consent forms.

## 3. Results

**Figure 1:**
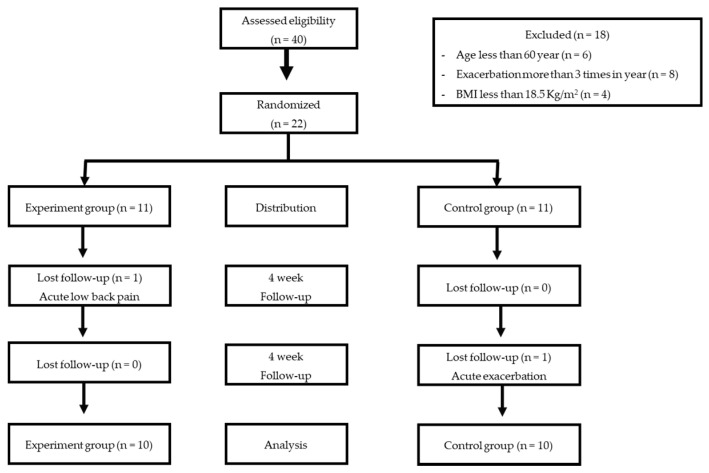
CONSORT flow chart of participant recruitment.

Forty participants joined the program, and eighteen were eliminated (age less than 60 years = six participants, exacerbation occurred over three times in a year = eight participants, excess BMI = four participants), leaving 22 participants left in the program divided into two groups of eleven. During the program, one participant of each group left due to acute exacerbation and back pain, leaving ten participants in each group to analyze (Figure 1). The characteristics of participants in both groups are shown in Table 2. Nineteen males and one female were included in this study. Forty percent of all participants smoked. There was no significant difference between groups in terms of age, height, weight, body mass index, level of lung obstruction, or cognitive assessment score.

This study found that the total CCQ score in both groups decreased significantly after the programs; however, there was no significant difference between the multimedia group and the control group. The physical performance according to the 6MWT showed no significant difference after the programs, and there was no significant difference between both groups. There was no significant difference in triceps strength muscle strength after the programs or between groups; however, quadriceps strength muscle strength demonstrated a significant difference after the multimedia rehabilitation program. Lastly, there was no significant change in quadriceps muscle strength between groups, as shown in Table 3.

**Figure 2:**
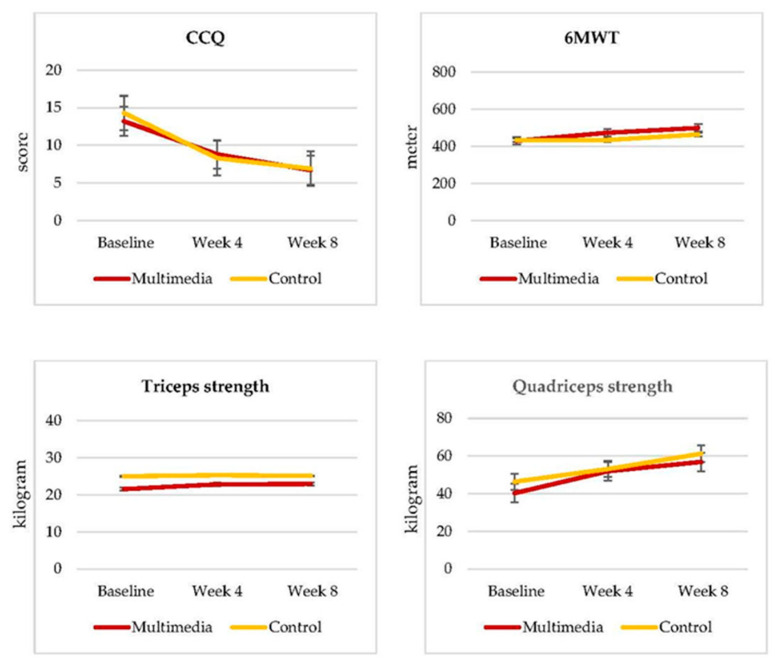
The trend results of clinical COPD questionnaire, 6 min walk test, and muscle strength of upper and lower extremities before and after the home-based pulmonary rehabilitation program between the multimedia and control groups.

In addition, there was no interaction shown between time and group for all parameters. The main effect of time was shown of CCQ (*p* < 0.001), 6MWT (*p* < 0.001), quadriceps muscle strength (*p* < 0.001). However, the main effect of time on triceps’ muscle strength (*p* < 0.338) was not demonstrated, as shown in Figure 2.

## 4. Discussion

An experimental study was performed to investigate the effect of a home-based pulmonary rehabilitation program with multimedia, including videos, flip charts, and a structural telephone monitoring evaluating clinical symptoms, exercise performance, and strength of the upper and lower extremities in patients with chronic obstructive pulmonary disease.

The participants of both groups were homogeneous (multimedia and control group were 69.20 ± 5.41 and 67.40 ± 6.60 years, respectively), and there were no differences in characteristics between groups. The weight and body mass index of both groups were within the normal range. The disease severity of both groups was at a moderate obstructive level. The cognitive evaluation scores of both groups were slightly lower than normal values [27]. Patients with chronic obstructive pulmonary disease have chronic hypoxia, which is chronic oxygen depletion that affects the anterior cerebral region, responsible for cognitive function and memory damage, thus causing problems in the learning processes, such as remembering and understanding [14,15]. For patients with cognitive impairment, the deficits in memory in COPD, programs might benefit from written information, visual cues, and involvement of a facilitating family member [17]. This study used multimedia in the form of videos and flip charts for stimulating all learning preferences of perception of patients in visual, auditory, reading, and kinesthetics of patients [28]. Structured telephone monitoring was used to stimulate motivation, review goals of the program, and evaluate and review problems with the program by a health professional [26]. In addition, this study facilitated family member involvement in supporting patients during the home-based pulmonary rehabilitation program. This factor could improve participant uptake of the program, especially in patients above 69 years old [8].

The results showed no significant difference in overall clinical evaluation scores between COPD patient groups (multimedia and control groups) before and after the home pulmonary rehabilitation program. The overall clinical symptom scores according to an assessment form showed a significant decrease in both groups after the program. The home pulmonary rehabilitation program helped to reduce symptoms, such as dyspnea, fatigue, and emotional stress, in addition to an improvement on the mMRC scale [11,26,29]. The exercise programs helped to work the muscles, improve respiratory function, decrease dyspnea, and increase functional activity [1,30,31]. Following the home pulmonary rehabilitation programs, there was a marked decrease in clinical symptoms, an increase in activity, and improved psychological scores. These positive results can encourage patients to undertake increased physical activity [1,29,32]. In addition, the weekly telephone monitoring increased patient adherence in both groups, thereby increasing the effectiveness of the home-based rehabilitation programs [26,32].

The results of the study showed that physical performance tended to increase, but there was no statistically significant difference in COPD patients provided home rehabilitation programs in the multimedia and control groups. These results may have stemmed from the fact that both groups of participants had received a continuous rehabilitation program prior to the study, including aerobic exercises, resistance exercises, breathing exercises, and physical therapy at the hospital on an individual basis. The 6 min walking distance was above the mean level of patients with chronic obstructive pulmonary disease without exacerbation, i.e., they were not at risk of hospitalization or death [33], while the level of intensity of the exercise in both groups of participants was moderate. The exercise program was a form of interval aerobic exercise combined with resistance exercises and chest trunk mobilization. It has been shown that providing exercise programs at an appropriate level of intensity for an individual increases their ability to exercise [34], whereas another study found that a high intensity can improve exercise capacity to a greater level than moderate and low intensities [35,36]. However, in this study, despite low to moderate exercise ability, there was a clinically significant improvement in 6 min walking distance after the home-based multimedia pulmonary rehabilitation program. The walking distance was increased to an average distance of 69.30 ± 53.45 m, which is clinically superior to the average of 35 m [37], whereas the control group increased their walking distance to an average of 30 m. This shows the clinical improvement of providing multimedia rehabilitation programs in patients with chronic respiratory disease.

The home-based rehabilitation program did not lead to a significant change in upper-limb muscle strength, whereas lower-limb muscle strength showed a statistically significant increase in the multimedia group and a tendency to increase in the control group (not significant). Previous studies found that the limb muscle strength in patients with chronic obstructive pulmonary disease was decreased in both the lower and upper extremities [38,39]. The size of the lower-limb muscles reflects their importance for functional activities. Thus, weakness in this area negatively affects a patient’s functionality and ability to perform various activities in daily life, as well as exercise [40,41]. Thus, the exercise programs administered target the leg muscles through both strengthening and aerobic exercises, showing satisfactory results [39,41]. In addition, demonstrative videos and flip charts helped patients in the multimedia group to improve the quality and quantity of exercise, thereby strengthening their muscles. The visual cue and auditory on video clarify the exercise position and movement during exercise. The written information and pictures on flip charts can help patients to correct the exercise when they are required. In addition, weekly structured telephone from a physiotherapist could help them if they had any questions about the exercise.

This study reflected an effect of a multidisciplinary home-based rehabilitation program, including exercise training, education in nutrition, medication management, smoking cessation, and self-management using instructive multimedia in the form of videos and flip charts. The results showed a trend of improvement of clinical symptoms, 6MWD, and strengthening of the quadriceps muscle. However, a significant difference between groups was not observed. It may be due to many factors, such as the level of exercise intervention. Therefore, the pulmonary rehabilitation program should be concerned with the aims of the program and concerned with education, support, and self-management according to objectives of the program for behavioral change and enhanced patient health. Patients have different learning styles and attention spans; therefore, the use of multiple modalities is preferred [18,19]. The previous study showed the effectiveness of a home-based pulmonary rehabilitation program using a booklet and manual. They also showed the improvement of physical performance and quality of life of patients [20,21]. Multimedia in the form of videos and flip charts for education and exercise on home-based pulmonary rehabilitation, for all learning preference stimulating perception, including visual, auditory, reading, and kinesthetics of patients, is feasible and accessible for elderly COPD patients with barriers to online communication.

This study had some limitations. Firstly, this research set the home-based exercise intensity according to a modified Borg scale score of 3–4, adjusted to the participants, which may be lower than suggested by the guidelines. However, this was to increase safety and improve patient compliance. Secondly, the multimedia group consisted of only one female participant, while there were no female subjects in the control group. However, the characteristics of the subjects were not significantly different between groups before intervention. Thirdly, the 8-week pulmonary rehabilitation program can be considered a short-term intervention. Thus, a long-term home-based multimedia pulmonary rehabilitation program should be evaluated in a future study.

## 5. Conclusions

This study demonstrated the effect of home pulmonary rehabilitation programs using multimedia (videos and flip charts), along with telephone monitoring. The multidisciplinary program included advice on disease, quitting smoking, nutrition, medication management, and self-management for chronic obstructive pulmonary disease, as well as interval exercise programs. The exercise program included aerobic exercises, resistance exercises, and chest mobilization for patients with chronic obstructive pulmonary disease. This program was found to reduce clinical symptoms, improve physical performance, and increase leg muscle strength in COPD patients.

Therefore, in COPD clinics, healthcare teams can provide home-based multimedia rehabilitation programs through stimulating patient perception with multiple learning preferences to promote physical performance and improve the symptoms of patients, especially in the elderly and/or cognitively impaired Chronic Obstructive Pulmonary Disease Patients.

## Figures and Tables

**Table 1:** Exercises included in the video.

Exercise video	Exercises	Repetitions or Time	Intensity
Free-load dynamic and stretching exercise	Warmup	2–3 min	Borg scale 1–2 “light”
	Pectoral muscles	30 s × 2	The point of feeling tightness or slight discomfort
	Triceps muscles	30 s × 2	
	Deltoid muscles	30 s × 2	
	Hamstring muscles	30 s × 2	
	Quadriceps muscles	30 s × 2	
	Gastrocnemius muscles	30 s × 2	
Resistance exercise	Pectoral muscles	2–3 sets	
using an elastic band	Triceps muscles	2–3 sets	
	Deltoid muscles	2–3 sets	10 RM
	Hamstring muscles	2–3 sets	
	Quadriceps muscles	2–3 sets	
	Gastrocnemius muscles	2–3 sets	
Aerobic exercise	On-the-spot marching alternated with chest trunk	10–40 min (Weeks 1– 8)	Borg scale 3–4 “moderate”
	mobilization (2 min–1 min)		
Free-load dynamic and stretching exercise	Cooldown	2–3 min	Borg scale 1–2 “light”
	Pectorals muscles	30 s × 2	The point of feeling tightness or slight discomfort
	Triceps muscles	30 s × 2	
	Deltoid muscles	30 s × 2	
	Hamstring muscles	30 s × 2	
	Quadriceps muscles	30 s × 2	
	Gastrocnemius muscles	30 s × 2	

RM, repetition maximum.

**Table 2:** Characteristics of participants.

Characteristics	Mean ± SD		
	Multimedia (*n* = 10)	Control (*n* = 10)	*p*-Value
Sex (%)			
Male	90	100	
Female	10	0	
Age (years)	69.20 ± 5.41	67.40 ± 6.60	0.513
Height (cm)	163.60 ± 6.17	165.90 ± 5.49	0.390
Weight (kg)	61.54 ± 9.83	59.78 ± 9.44	0.688
Body mass index (kg/m^2^)	22.94 ± 3.05	21.63 ± 2.53	0.308
FEV_1_ (% predicted)	64.60 ± 11.97	60.40 ± 20.14	0.578
MoCA score	22.40 ± 2.88	24.40 ± 2.46	0.112

SD, standard deviation; FEV_1_, force expiratory volume in 1 s.

**Table 3:** Results of clinical COPD questionnaire, 6 min walk test, and muscle strength of upper and lower extremities before and after the home-based pulmonary rehabilitation program between the multimedia and control groups.

Variables	Mean ± SD		
	Multimedia (*n* = 10)	Control (*n* = 10)	*p*-Value
**CCQ**: (score)			
Baseline	13.20 ± 4.87	14.30 ± 8.51	0.727
Week 4	8.80 ± 3.74	8.30 ± 3.09	0.748
Week 8	6.70 ± 3.71 ^a^	6.90 ± 5.59 ^a^	0.926
%Change	−6.50 ± 4.86	−7.40 ± 7.26	0.749
**6MWT**: (meter)			
Baseline	429.65 ± 63.42	434.16 ± 56.25	0.868
Week 4	473.20 ± 58.57	434.45 ± 61.61	0.167
Week 8	499.00 ± 78.60	464.40 ± 41.12	0.233
%Change	69.30 ± 53.45	30.20 ± 36.06	0.073
**Triceps strength**: (kg.)			
Baseline	21.50 ± 6.54	24.95 ± 11.12	0.226
Week 4	22.80 ± 4.83	25.25 ± 10.19	0.504
Week 8	22.90 ± 6.90	25.10 ± 13.29	0.648
%Change	2.60 ± 5.60	0 ± 6.20	0.733
**Quadriceps strength**: (kg.)			
Baseline	40.25 ± 6.98	46.35 ± 12.19	0.191
Week 4	51.95 ± 13.46	53.10 ± 15.79	0.863
Week 8	56.90 ± 10.49 ^a^	61.25 ± 13.11	0.423
%Change	16.50 ± 6.19	14.90 ± 13.14	0.338

Kg., kilogram; CCQ, clinical COPD questionnaire; 6MWT, 6 min walk test; SD, standard deviation; ^a^ significant difference before and after rehabilitation program, *p* < 0.05.

## Data Availability

All data are reported in the study.
